# The Effects of Imitative Vs. Cognitive Methods on The Speech Development of Children With Autism

**Published:** 2014

**Authors:** Monireh JALILI, Nader JAHANGIRI, Amir Amin YAZDI, Farah ASHRAFZADEH

**Affiliations:** 1 Department of Linguistics, Faculty of Letters and Humanities, Ferdowsi University of Mashhad, Mashhad, Iran; 2 Faculty of Psychology, Ferdowsi University of Mashhad, Mashhad, Iran; 3 Pediatric Neurology Department, Faculty of Medicine, Ghaem Hospital, Mashhad, Iran

**Keywords:** Autism, Imitative method, Cognitive method, Speech development

## Abstract

**Objective:**

The present study was performed to examine the effects of two speech therapy methods on six verbal behaviors of autistic children, including oral speech, listening, organizing, speaking, semantics, and syntax.

**Materials & Methods:**

In this study, thirty children with autism were assigned to one of two groups: imitative and cognitive groups. Before starting the main procedures of the study, the children of both groups were homogenized concerning their autism level. In the first phase of the study, the speech development level of the two groups was measured in a pre-test, in which both groups showed similar results. Then, both groups of children received 6 months of speech therapy instruction, during which one group was taught using an imitative method, while the other group was being worked with cognitive method.

**Results:**

After 6-month treatment period, a post-test was done, and the t-tests based on the data of the two groups revealed a significant difference between the results.

**Conclusion:**

The statistics showed that after the teaching period, autistic that worked with cognitive method gained a better development in their speech abilities, comparing to those worked with the imitative method.

## Introduction

Autism is a complicated neurological disorder, which impairs patient`s ability to socialize, communicate, process of sensory information, and experience of the full spectrum of interests common to most people. Today autism spectrum disorders (ASDs) may also be recognized with other terms, such as “classic autism”, “kanner’s autism”, and “Asperger syndrome” (1).

Recently, the number of people with ASD has been decreasing noticeably. In the last two decades, 5-15 in every 10000 births were reported to be affected by autism. Today, the center of recognition and disease control believes the ASD spreading is one in every 166 births in general (2). 

Fombonne (2005) believes that 60–70 in every 10000 children are born with ASD. This number has been reported differently for different countries (3). Cook et al. (4) mentioned 43.1 per 10000 for Canada, using the state data in Ministry of Education. In Iran, Samadi (2008) reported 190 in 10000 in Shiraz, one of the biggest cities of Iran (5).

Children with ASD have many problems in different areas of language speaking and communication. Communicating with others makes the child able to begin social interactions, but children with ASD lack the ability to communicate effectively. This deficiency is considered as one of the prominent factors of these kinds of disorders. The importance of early speech therapy for young children with autism is obvious to everyone. Rogers (1999) found out that some kinds of early interventions help children with autism to be successful much more quickly comparing to those with other neurodevelopmental disorders (6).

Pennington and Rogers (1991) found that imitation is one of the relevant issues related to autism, because it affects social cognitions like that of theory of mind, empathy, and speech development (7). Impairments in these abilities characterize individuals with ASD, which has caused some theorists to suggest that an imitation impairment is the core deficit in patients with ASD (8). Even if one’s with ASD have a functional mirroring deficit, It is highly improbable that this would be enough to explain why they perform poorly in most imitation tests. Imitative performance typically includes a wide range of cognitive, motivational, and praxic abilities, involving perceptual processing of complex stimuli, attentional control, executive function, motor control, theory of mind, language, and the comprehension of social cues (6,9). Several studies have applied modeling as a means of teaching language, affective communication, and social skills to autistic children, in spite of their underdeveloped imitative repertoires (10), and some researchers have counted on imitation as a prerequisite to the learning of more complex responses like spontaneous language (11).

Cognition is also one of the important issues related to speech therapy treatments, which starts with imitation, and when imitation phase is fixed, it will continue on more details (12) In 1998, De Giacomo and Fombonne (13) who carried out a study in European countries, found out that cognition is very important in teaching children with autism, and helps autistic children who have had some delays in their speech. Hodgdo (2001) has also mentioned the cognitive method in her book, as one of the useful interventions that can be used to help autistic children in their communication skills (14). In 2001, berterand et al. (15) did a research on cognition in American societies. Since having problems in imitative skills are common among children with autism, some researchers have relied on cognition in their studies (16,17). Most of these works have been done on the behaviors rather than the speech development in autistic children. There is no related literature about using cognitive methods in speech therapy for autistic children in Iran, and thus, this study is the first of its kind in this country. 

It is also worth mentioning that although some researchers such as Ghanizadeh (2008) and Samadi (2001) have done some studies on the spreading amount of autism in Iran, similar researches related to areas of imitation and cognition in speech therapy for children with ASD has not been done previously in Iran, and imitative method is an old traditional method widely used in speech therapy clinics of this country. 

## Materials & Methods

**Participants**

A total of 30 participants, including two groups of 15 children with ASD were examined. Participants` age ranged from 6 to 8 years old (mean age=7.5 years), and they were composed of 12 girls and 18 boys (gender was not a considered variable in this study). Before conducting the main stage of the program, a CARS (Childhood Autism Rating Scale) test (18) was used to homogenize the participants in the two groups concerning their autism level. CARS has been shown to have 100% predictive accuracy when distinguishing autism between groups of autistic and intellectually disabled children (19).

The statistics are as follows:

**Table1:** The Autism Level of The Two Groups

**Group Statistics**				
**N**	Mean	Mode	Standard deviation	Range
30	40.175	39.0	5.08	19.5

Childhood Autism Rating Scale (CARS) test is composed of 15 questions about the behaviors of children, that rates them on a scale from one to four for various criteria, ranging from normal to severe, and producesa composite score that ranges from nonautistic (15) to mildly autistic (15-30), moderately autistic (30-50), or severely autistic (50-60). The mean score of children in both groups was 40.175, which shows all of them were moderately autistic children. Also, three other children whose scores were not in the range of the study were excluded from the participants in order to have homogeneous groups.

**Procedure**

This study was composed of a pre-test and a post-test. The epidemiological procedure of this study started from January 2012 to August 2012. Initially, 30 Autistic children were selected for the experiment, and divided into two groups (without considering their gender), according to the speech therapy treatments they were about to receive. All children had received a Diagnostic and Statistical Manual-IV (DSM-IV) diagnosis of autism. Before starting the main procedure, we also used CARS test to measure the autism level of the participants, and the two groups did not differ on this variable, showing the equal level of autism (mean=40.175). Then, a pre-test (T1) was done before the start of treatments, and the results were recorded. After the T1, both groups underwent a 6-month period of speech therapy treatment. One of the groups was given the imitative treatment of speech therapy, while the other underwent the cognitive method. At the end of the 6-month period of treatment, a post-test (T2) was taken from both groups to measure the gains the participants had made. The results were recorded once more, and compared to those of T1. The statistics were then checked and analyzed by a statistician.

**Tools**

In this study CARS test was used to measure the autism level of the participants. The answer sheets of this test were completed by the parents and the speech therapist for each child. The test used for the original study, was the test of language development (TOLD) (20), which was completed by the researcher for each participant. The TOLD test includes 9 subtests, which by combining their results together, 6 final scores (oral speech, listening, organizing, speaking, semantics, and syntax) are obtained.

**Treatment**

At the first confrontation with children, the effort was made to communicate effectively with children during their playtime through playing and talking with them. During the treatment period, each participant in both groups received two sessions of speech therapy a week, and each session lasted for 30 minutes. Group A underwent imitative method, and group B took part in the cognitive method. These speech therapy treatments were held in a normal room equipped with two chairs, a table, and some picture cards to teach the new words. The procedure for teaching new words via the imitative method included modeling, prompting, and error correction. Thus, the speech therapist uttered a word (cow for example) for two or three times pointing to the related picture card, and then used the same card to ask the child the name of that picture. In this method, no more details (such as the color of a cow, its sound, its food, etc.) were explained to the child. 

In this method, the child learns to imitate and repeat whatever he/she hears (21). This is a traditional 

method widely used in most of the speech therapy clinics of Iran.

**Table 2:** Comparison of The Scores of The Two Groups In T1

		**N**	**Mean**	**Median**	**Mode**	**Range**	**Variance**	**SD**	**T-value**
**Oral speech**	Group A	15	95.5	102	92	44	132.02	11.49	0.87
	Group B	15	99.2	97	87	32	92.36	9.08	
**Listening**	Group A	15	97.13	99	105	26	61.98	7.87	0.939
	Group B	15	94.33	97	97	33	71.15	8.43	
**Organizing**	Group A	15	97.66	103	103	61	196.88	14.03	1.504
	Group B	15	104.26	100	98	33	91.92	9.58	
**Speaking**	Group A	15	97.6	97	97	42	90.86	9.53	0.380
	Group B	15	98.4	92	89	32	111.98	10.58	
**Semantics**	Group A	15	103.8	108	109.1	30	66.02	8.1	0.36
	Group B	15	104.9	102	102	35	79.12	8.8	
**syntax**	Group A	15	90.2	95	95	56	182.69	13.51	0.122
	Group B	15	90.7	89	85	34	101.12	10.05	

The second group received the cognitive speech therapy treatments. The cognitive method starts with imitation, and after the imitation phase is completely fixed, more linguistic samples, details, and questions are presented to the children for cognition. So, the speech therapist starts the treatment process similar to that of imitative method, and after the child managed to repeat and imitate the learnt word accordingly, the speech therapist goes through the second phase of teaching, including giving other details about the word and asking different questions. 

In this level, the speech therapist first starts with yes/ no questions, and after he becomes quiet assured about the fixation of the word`s concept in the mind of the child, he starts to ask information questions like (what, where, when, how, etc.) in order to make the child`s mind explore and think more about what he/she has learnt. Also, the child has to answer some questions, in which the order of the words has been changed (12). The child is also asked to make sentences about the picture he/she is seeing, and give much more explanations. The child is also encouraged to utter the sentences distinctively.

## Results

**1. Comparison of the scores of two groups in T1**

The TOLD test was used to analyze the differences in speech between the two groups. The results show that there were no significant differences between the speech abilities of the groups in the first phase of the research. Since the T-values for the oral speech (0.87), listening (0.939), organizing (1.504), speaking (0.380), semantics (0.36), and syntax (0.122), are all less than the T-value for the degree of freedom of 28, (p<0.0= 2.46), thus the differences between the two groups are not significant, and it shows that during T1, there were no differences in these skills between the two groups.

**2. Comparison of the scores of the two groups in T2**

The second analysis was done based on the results of the post-tests the two groups had taken. This analysis showed that after 6 months of speech therapy, the two groups showed different results. In this analysis, the results revealed that group B, which had received the cognitive speech therapy treatment, had more improvement and showed better scores compared to group A, which had worked with an imitative method during the treatment.

**Table3:** Comparing of The Scores Between The Two Groups In T2

		**N**	**Mean**	**Median**	**Mode**	**Range**	**Variance**	**SD**	**T-value**
Oral speech	Group A	15	110	111	111	48	132.53	11.512	2.97
	Group B	15	122.6	117	117	38	137.3	11.717	
Listening	Group A	15	108	110	117	27	78.93	8.88	3.206
	Group B	15	120.2	125	110	36	138.16	11.75	
Organizing	Group A	15	105.6	108	108	39	95.173	9.75	4.74
	Group B	15	123.26	119	119	32	112.86	10.62	
Speaking	Group A	15	104.46	103	103	31	59.91	7.74	6.208
	Group B	15	121.6	121	121	31	68.24	8.26	
Semantics	Group A	15	110.8	113	113.120	27	56.38	7.58	4.41
	Group B	15	125.53	120	117	30	109.44	10.461	
syntax	Group A	15	101.26	105	105	47	116.46	10.79	3.854
	Group B	15	116.66	112	105	36	123.02	11.091	

The post-test T-values of the two groups in oral speech (2.97), listening (3.206), organizing (3.206), speaking (3.206), semantics (4.41), and syntax (3.854) are more than the T-value for the 28 degree of freedom (p<0.01=2.46). It shows that the differences between the two groups are significant, thus the feedbacks of the groups were totally different, and not caused by chance. 

Considering the means of these scores after 6 month of treatment, although both groups had progressed, group B was noticeably better in the tested skills, compared to group A.

**3. Comparison of the scores of two groups in T1 and T2**

In the third analysis, T1 scores of both groups were compared to T2 scores of them to show their amount of progress made by the 6 months of treatment. The results showed although both groups had progressed after the treatment, patients of group B, who had received the cognitive speech therapy treatment, showed a better improvement in reaching language skills. 

A matched T-test was used to compare the means of T1 and T2 of each group. Thus, this time the degree of freedom was 14, and the T-value for this degree of freedom is 2.62. (p<0.01= 2.62) (22). Also, the T-values less than 0.01 shows that the result of only 1 out of 100 cases, might be due to chance (23,24).

The comparison of t-tests of 6 speech development skills between the two groups was separately performed during T1 and T2, and are as follows:

**1-1. Oral speech**

**Table 4:** Comparison of T-Values In Oral Speech

		**N**	**Mean**	**Median**	**Mode**	**Range**	**Variance**	**Standard deviation**	**T-value**
**Group A**	T1	15	95.8	102	92	44	132.02	11.48	3.38
	T2	15	110	111	111	40	132.53	11.512	
**Group B**	T1	15	99.2	97	87	32	92.56	9.62	5.977
	T2	15	122.6	117	117	38	137.3	11.71	

The T-values for oral speech in group A (3.38), and group B (5.977) were more than the T-value for the 14 degree of freedom. It shows both groups have progressed significantly after the treatment, but group B showed much more improvement in oral speech.

**1-2. Listening**

**Table 5:** Comparison of T-values In Listening

		**N**	**Mean**	**Median**	**Mode**	**Range**	**Variance**	**Std. deviation**	**T-value**
**Group A**	T1	15	97.13	99	105	26	61.98	7.78	3.545
	T2	15	108	110	117	27	78.93	8.88	
**Group B**	T1	15	94.33	97	97	33	71.15	8.43	6.924
	T2	15	120.2	125	110	36	138.16	11.754	

The T-values for listening in group A (3.545) and group B (5.924) were more than that for the 14 degree of freedom.

It shows that both groups have progressed significantly after the treatment, Although group B showed a better improvement in the listening skill.

**1-3. Organizing**

**Table 6:** Comparison of T-Values In Organizing

		**N**	**Mean**	**Median**	**Mode**	**Range**	**Variance**	**Std. deviation**	**T-value**
**Group A**	T1	15	97.66	103	103	61	196.88	14.03	1.797
	T2	15	105.6	108	108	39	95.173	9.75	
**Group B**	T1	15	104.26	106	98	33	91.92	9.58	4.955
	T2	15	123.26	119	119	32	112.86	10.62	

The T-value for organizing in group A (1.797) is less than the that of the 14 degree of freedom. So, the amount of progress between T1 and T2 for group A was not significant. The T-value for group B (4.955) was more than that for the 14 degree of freedom. It shows both groups have progressed significantly after the treatment, but group B showed a better improvement in organizing.

**1-4. Speaking**

**Table 7:** Comparison of T-Values In Speaking

		**N**	**Mean**	**Median**	**Mode**	**Range**	**Variance**	**Standard deviation**	**T-value**
**Group A**	T1	15	97	97	103	42	90.86	9.53	2.3901
	T2	15	104.46	103	103	49	59.91	7.74	
**Group B**	T1	15	98.46	92	92	32	111.98	105.8	5.506
	T2	15	121.6	121	121	31	68.24	8.26	

The T-value for speaking in group A (2.3901) is less than that for the 14 degree of freedom. So, the amount of progress between T1 and T2 in group A was not significant. The T-value for group B (5.506) was more than that for the 14 degree of freedom. It shows that both groups have progressed significantly after the treatment, but group B showed a better improvement in speaking.

**1-5. Semantics**

The T-value for semantics in group A (2.47) is less than thatfor the 14 degree of freedom. Thus, the amount of progress between T1 and T2 for group A was not significant. The T-value for group B (5.809) was more than that for the 14 degree of freedom. Therefore, both groups have progressed significantly after the treatment, but group B showed a better improvement in semantics.

**1-6. Syntax**

The T-value for syntax in group A (2.47) is less than that for the 14 degree of freedom. So, the amount of progress between T1 and T2 for group A was not a significant one. The T-value for group B (6.708) was more than that for the 14 degree of freedom. Thus, both groups have progressed significantly after the treatment, and group B showed a better improvement in syntax.

The overall progress of the two groups can be seen in the following figures:

**Fig 1:**
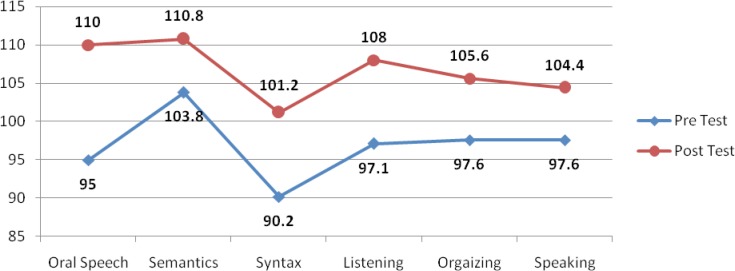
The progress of speech abilities in group A

As it can be seen in this diagram, group A had better scores in the T2 of all of the 6 skills tested in this research comparing to its T1, which shows the progress of this group after the treatment period.

**Fig 2:**
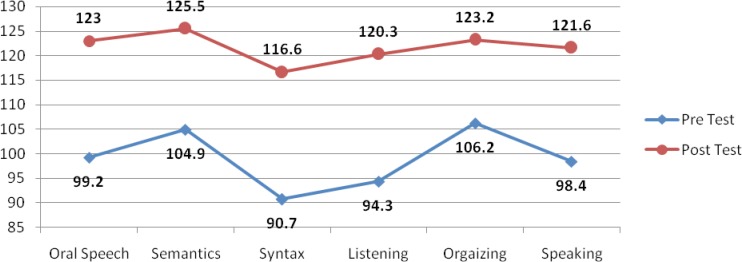
The progress of speech abilities in group B

**Fig 3:**
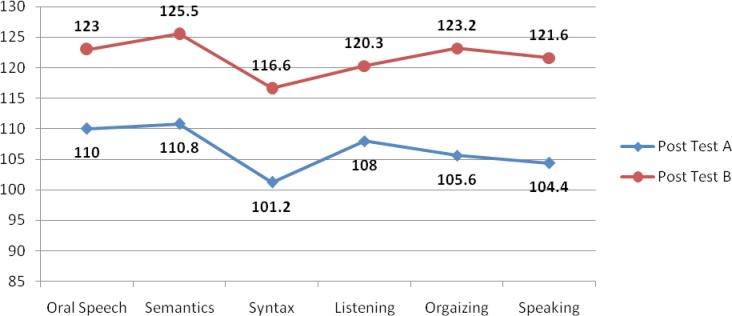
Comparison of group A and B in T2

It is also seen in the diagram that after the period of treatment, group B has also progressed in the above mentioned skills in T2 compared to T1. 

Overall comparison of both groups revealed that group B did better than group A after the 6 months of speech therapy treatment.

Comparison of the scores of both groups showed more progress of speech development in group B, as it is shown in the diagrams based on the statistics.

**In conclusion**, the present study investigated the language skills and their progress level in autistic children during two phases, between which there was a 6-month period of speech therapy treatment. During T1, the children in both groups did not show any significant differences in the test, and their scores were the same. After T1, the children participated in a 6-month speech therapy treatment. The effects of two speech therapy methods on the speech development of autistic children then measured and analyzed. T-tests yielded differences between the two groups. These differences showed the scores of group A that had received the imitative method, were increased, but this increase was not as much as the scores of group B. Matched T-tests showed group B had a better improvement in all of the sub tests including oral speech, listening, organizing, speaking, semantics, and syntax. 

Overall, these results demonstrate the effectiveness of cognitive speech therapy method over the imitative method in the speech development of children with autism. The traditional imitative method is still used in many of the speech therapy centers, but according to the results found in this study, it is obvious that replacing this method with the cognitive method can be more effective in helping speech problems of autistic children.

Since the children in the imitative method group had also shown a little progress in their speech abilities, it can be concluded that this method can be somehow effective. Therefore, the speech therapy can be started with an imitative phase, and then be continued with a cognitive phase.
